# Acute Stridor and Respiratory Failure due to Retrosternal Subglottic Stenosis of Unknown Origin

**DOI:** 10.1155/2013/728405

**Published:** 2013-07-15

**Authors:** Tharindu Vithanage, Gerben Keijzers, Nicola Jane Willis, Tara Cochrane, Linda Smith

**Affiliations:** ^1^Intern, Gold Coast Hospital, Southport QLD 4215, Australia; ^2^School of Medicine, Bond University, Gold Coast QLD 4226, Australia; ^3^Department of Emergency Medicine, Gold Coast Hospital, Southport QLD 4215, Australia; ^4^School of Medicine, Griffith University, Gold Coast QLD 4215, Australia; ^5^Intensive Care Unit, Gold Coast Hospital, Southport QLD 4215, Australia; ^6^Haematology Department, Gold Coast Hospital, Southport QLD 4215, Australia; ^7^Anaesthetics Department, Gold Coast Hospital, Southport QLD 4215, Australia

## Abstract

Respiratory failure due to subglottic stenosis is a rare but serious condition. A 22-year-old male presented to the emergency department (ED) with shortness of breath, stridor, and change in tone of voice. The patient did not complain of B-symptoms (fever, weight loss, and night sweats). In the week before this presentation, he was diagnosed with an upper respiratory tract infection with associated bronchospasm and discharged on oral antibiotics and inhaled salbutamol without effect. He developed hypercapnic respiratory failure in the ED after a coughing episode. A normal nasopharyngoscopic examination and a subtle mediastinal abnormality on chest radiograph lead to a working diagnosis of retrosternal subglottic obstruction. The complexities of his airway management and suggestions for multidisciplinary approach are discussed.

## 1. Introduction

Respiratory failure due to subglottic stenosis is a rare but serious condition. There are no guidelines for distal, retrosternal airway stenosis.

## 2. Case Report

A 22-year-old man of Mediterranean descent presented by ambulance to the ED complaining of intermittent coughing associated with severe shortness of breath, stridor, and change of voice tone. Two episodes of altered level of consciousness, associated with incontinence and cyanosis, were reported prehospitally before paramedic assessment. On arrival in ED, he was alert, with oxygen saturations of 88% on room air and a respiratory rate of 60. Further history revealed that he had been assessed in two other EDs in the preceding fortnight and discharged with a working diagnosis of upper respiratory tract infection with associated bronchospasm. He was discharged 4 days previously on oral roxithromycin and inhaled salbutamol. There was no improvement of symptoms since and he suffered paroxysmal coughing episodes triggering severe dyspnoea. He had no significant past medical history and he did not take any regular medications. He was a nonsmoker, who denied any illicit drug use. He was a pharmacy student with no relevant family history. 

After initial assessment in ED, his respiratory parameters improved to an oxygen saturation of 99% on room air and a respiratory rate of 26/min; however, a repeat episode of coughing again lead to diaphoresis, stridor, and respiratory rate of 60/min with oxygen saturations of 88% on room air. High-flow oxygen was administered and an arterial blood gas was taken, which showed acute hypercapnic respiratory failure (PH: 7.17, PCO_2:_ 77, PO_2:_ 102, and HCO_3:_ 27).

**Figure 1:**
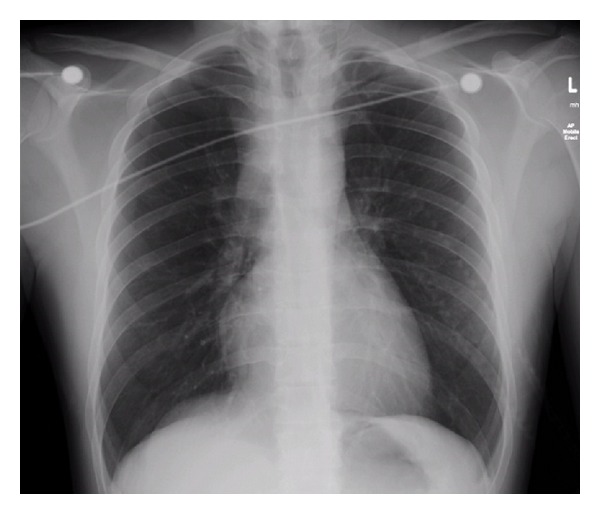
CXR on presentation.

The patient was treated with nebulised adrenalin and urgent nasopharyngoscopy was performed by the Ear, Nose and Throat (ENT) registrar, who could not detect any obstruction at or above the glottis. Review of the chest radiograph (CXR) prompted consideration of tracheal stenosis above the carina (Figure 1).

Since the location of the stenosis was retrosternal and etiology and exact size of suspected tracheal narrowing was uncertain, airway management of this patient required careful planning and multidisciplinary discussion (ED, ICU, anaesthetics, ENT, and thoracic surgery). Although awake fibre-optic intubation was discussed and considered, there was considerable reluctance to intubate the patient since the narrowing could be either highly vascular or friable in nature with subsequent complications. Also, a fibre-optic scope could potentially cause complete obstruction with limited ability to “rail-road” an endotracheal tube. It was decided that a CT scan (to delineate location and nature of mass) before definitive airway management was desired, which resulted in a trial of noninvasive ventilation (NIV) using bilevel positive airway pressure (BiPAP) with the patient in a supine position for 10 minutes in the ED. The patient tolerated this well, and a CT scan was performed with the patient on NIV.

**Figure 2:**
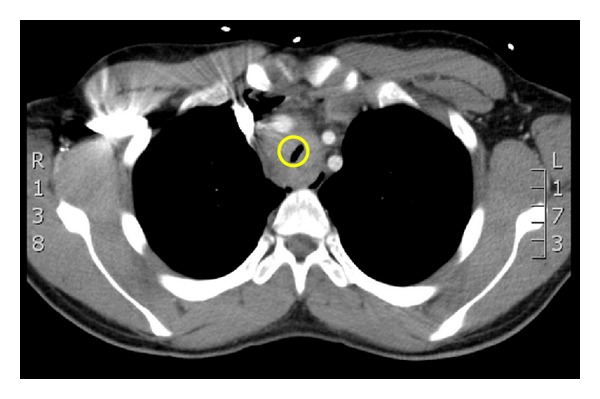
Transverse CT.

The CT showed a circumferential mass surrounding the trachea causing stenosis at 3–6 cm above the carina with a minimum lumen diameter of 2-3 mm (Figure 2). The mass originated from the mediastinum, and exact dimensions were difficult to measure and were estimated to be 8 × 5 × 4 cm. The patient was subsequently intubated in theatre under gas induction (with the patient spontaneously breathing and sitting up), using a size 6 microlaryngoscopy tube (MLT) in the presence of an ENT surgeon and thoracic surgeon. There was transient hypoxia after right endobronchial intubation, which was promptly rectified by adjusting the tube depth. A surgical biopsy of the mediastinal mass compressing the trachea was taken. The histology was consistent with nodular sclerosing Hodgkin's Lymphoma. Therapy with high-dose dexamethasone (40 mg IV per day) in ICU did not improve the airway stenosis. On his 3rd day in ICU, the patient received his first dose of chemotherapy with adriamycin, bleomycin, vinblastine, and dacarbazine (ABVD) while intubated and ventilated on room air. This resulted in a rapid improvement in the airway stenosis—resulting in a cuff leak—allowing a larger endotracheal tube to be inserted initially, then subsequent extubation six days after chemotherapy. The patient went on to complete four cycles of ABVD chemotherapy and consolidative radiotherapy to the mass. Fourteen months after the original presentation, the patient was in remission and has resumed his studies.

## 3. Discussion

This case highlights the challenges of the airway management of a patient with respiratory failure due to a retrosternal subglottic stenosis of unknown origin. A CT scan was required for further airway assessment and diagnosis, but was challenging to obtain without definitive airway management. Multidisciplinary discussion amongst the consultant staff from the involved specialties led to the described course of action.

One of the most challenging situations in emergency medicine is the “cannot intubate, cannot ventilate” scenario. Several algorithms exist [1–4] to assist the clinician in the approach to the difficult airway, which include a surgical airway or cricothyroidotomy. However, in our reported case, a surgical airway is not likely to be beneficial since the stenosis is suspected to be below the sternal notch (in the mediastinum). For this scenario, there is no consensus approach or guideline. The uncertainty regarding the etiology of the subglottic mass and subsequent possible complications made a consensus decision regarding airway management difficult. The disciplines involved discussed the relative benefits and disadvantages of (fibre-optic) intubation before CT or the use of NIV to facilitate the gaining of further information (i.e., CT scan). It was decided to elect for the latter option, although they acknowledged that this approach was not ideal, since the risk of potential loss of airway in an uncontrolled environment was not negligible. The patient was accompanied with appropriately skilled consultant staff and equipment to the CT scanner. 

There are no clear airway algorithms for (suspected) distal or retrosternal subglottic stenosis of unknown origin. Most common difficult airway algorithms [4, 5] advocate rapid sequence intubation if the standard algorithm fails; however, in this case where location and nature of the mass are uncertain and the patient is still awake and conscious, other strategies must be considered.

Most of the (ENT and anaesthetics) literature on the management and approach to subglottic stenosis relates to previously known and congenital stenosis [6–8]. The approach for the management of subglottic stenosis in the emergency setting often involves cricothyrodotomy [9]. This is only useful if the subglottic stenosis is above the cricothyroid membrane or at least above the lowest tracheal ring above the sternal notch. This was not the case in our patient.

## 4. Suggested Approach

**Figure 3:**
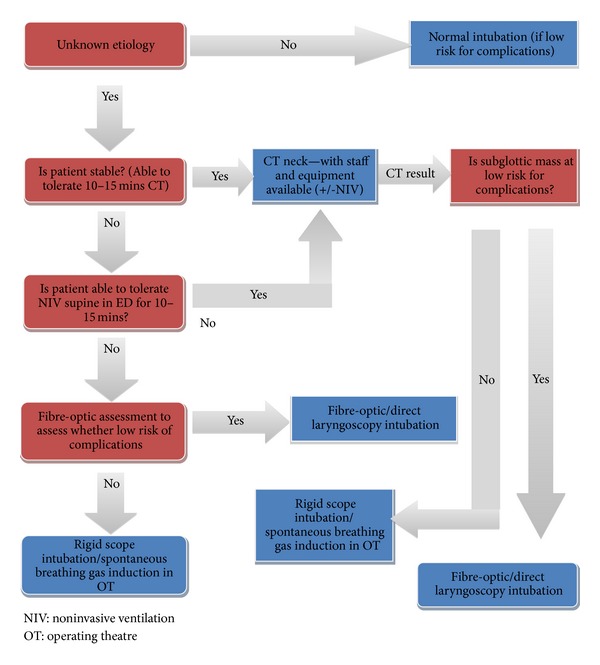
Difficult airway algorithm—suspected subglottic stenosis of unknown etiology.

We suggest that in a patient with subglottic stenosis of unknown etiology who is in distress, the strategy should be determined by the experience of available staff and equipment available (see Figure 3—flow diagram). Suggested strategies include (1) awake fibre-optic assessment—if the risk of intubation after visualizing the mass is deemed low, this can be attempted using either direct or video larnygoscopy or fibre-optic intubation. If the risk is moderate or high when the mass is visibly high in vascularity or friable, either a spontaneously breathing gas induction or rigid bronchoscopy can be used to secure the airway; (2) trial of supine NIV in the ED—if the patient tolerates this for 10–15 minutes, the CT can be performed on NIV with appropriate staff and equipment available. Both scenarios should have a multidisciplinary approach including ED, ENT, anaesthetics, ICU, and thoracic surgery involvement.

## 5. Conclusion

Emergency physicians should articulate an approach for (retrosternal) subglottic stenosis as part of the difficult airway algorithm.
